# Digital colloid-enhanced Raman spectroscopy for the pharmacokinetic detection of bioorthogonal drugs†

**DOI:** 10.1039/d4sc02553a

**Published:** 2024-08-02

**Authors:** Xinyuan Bi, Zhicheng He, Zhewen Luo, Wensi Huang, Xingxing Diao, Jian Ye

**Affiliations:** a State Key Laboratory of Systems Medicine for Cancer, School of Biomedical Engineering, Shanghai Jiao Tong University Shanghai 200030 P. R. China yejian78@sjtu.edu.cn; b Shanghai Institute of Materia Medica, Chinese Academy of Sciences Shanghai 201210 P. R. China; c University of Chinese Academy of Sciences Beijing 100049 P. R. China; d Institute of Medical Robotics, Shanghai Jiao Tong University Shanghai 200240 P. R. China; e Shanghai Key Laboratory of Gynecologic Oncology, Ren Ji Hospital, School of Medicine, Shanghai Jiao Tong University Shanghai 200127 P. R. China; f Sixth People's Hospital, School of Medicine, Shanghai Jiao Tong University Shanghai 200233 P. R. China

## Abstract

Bioorthogonal drug molecules are currently gaining prominence for their excellent efficacy, safety and metabolic stability. Pharmacokinetic study is critical for understanding their mechanisms and guiding pharmacotherapy, which is primarily performed with liquid chromatography-mass spectrometry as the gold standard. For broader and more efficient applications in clinics and fundamental research, further advancements are especially desired in cheap and portable instrumentation as well as rapid and tractable pretreatment procedures. Surface-enhanced Raman spectroscopy (SERS) is capable of label-free detection of various molecules based on the spectral signatures with high sensitivity even down to a single-molecule level. But limited by irreproducibility at low concentrations and spectral interference in complex biofluids, SERS hasn't been widely applied for pharmacokinetics, especially in live animals. In this work, we propose a new method to quantify bioorthogonal drug molecules with signatures at the spectral silent region (SR) by the digital colloid-enhanced Raman spectroscopy (dCERS) technique. This method was first validated using 4-mercaptobenzonitrile in a mixture of analogous molecules, exhibiting reliable and specific identification capability based on the unique SR signature and Poisson-determined quantification accuracy. We further developed a single-step serum pretreatment method and successfully profiled the pharmacokinetic behavior of an anticancer drug, erlotinib, from animal studies. In a word, this method, superior in sensitivity, controllable accuracy, minimal background interference and facile pretreatment and measurement, promises diverse applications in fundamental studies and clinical tests of bioorthogonal drug molecules.

## Abstract 2

Bioorthogonal drug molecules with the Raman signatures at the spectral silent region have been quantified with controllable reproducibility by digital colloid-enhanced Raman spectroscopy for rapid pharmacokinetic detection in live animals.

## Graphical abstract

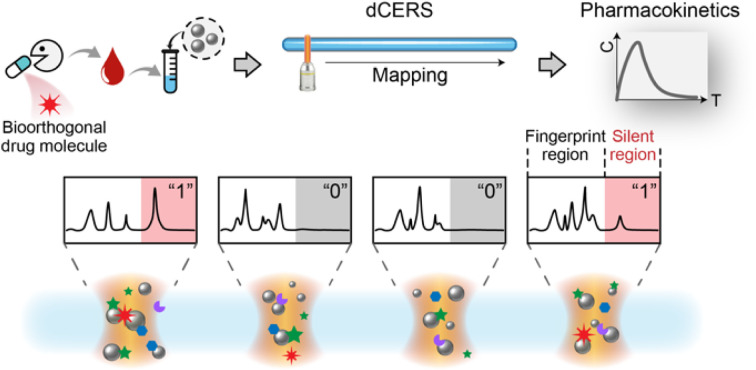

## Introduction

In recent years, a wide range of molecules with specialized functional groups have been under rapid development, particularly in the realm of drug molecules with several exogenous moieties such as nitriles, deuterium, alkynes, *etc.* Nitrile, a commonly used functional group in drug molecules, is present in approximately 2.4% of 2327 marketed drug molecules in the DrugBank, such as escitalopram, verapamil, rilpivirine, teriflunomide, vildagliptin, tofacitinib, among others.^1,2^ It is employed to enhance the bioavailability, selectivity, and binding affinity to the target proteins, and metabolic stability.^3,4^ Ongoing research explores new nitrile-functionalized molecules, *e.g.*, borrelidin^5^ and dihydroquinopimaric acid derivatives with nitrile groups.^6^ Deuterium plays a crucial role in extending a drug's half-life in the body, resulting in improved exposure profiles and reduced toxic metabolites, thereby enhancing efficacy and safety.^7,8^ Examples include the first FDA approved deuterated drug, deutetrabenazine in 2017,^9^ and deucravacitinib in 2022.^10^ Alkynes are commonly found in drug molecules, promoting good compatibility,^11^*e.g.*, efavirenz, norgestrel, ethinyl estradiol, *etc.* As these drugs flourish, a comprehensive understanding of their biological and physiological mechanisms becomes crucial for tailored therapeutic approaches.

Pharmacokinetic study, which is performed to monitor the drug concentration in the body, reflects how the body interacts with the drug upon the whole duration of exposure including drug adsorption, distribution, metabolism and elimination/excretion.^12^ For a given drug candidate, its pharmacokinetic properties play an indispensable role in drug discovery and development.^13,14^ By investigating the pharmacokinetic principles, prescribers can be informed of the dynamic drug effects and adjust the dose for individualized pharmacotherapy more accurately and in time.^15,16^ Current pharmacokinetics studies usually involve liquid chromatography-mass spectrometry (LC-MS) or high-performance LC-MS,^17–19^ which, despite their effectiveness, face challenges with regard to high expense, large instrumentation, complex pretreatment procedures and need for considerable expertise.^20,21^ In the case of pharmacokinetic detection in some clinical applications such as point-of-care test, state-of-the-art techniques should be preferred with small instrumentation, fast and simple pretreatment as well as operability by clinicians with only basic training.^17,22,23^

SERS is a fingerprinting vibrational spectroscopy which is capable of label-free detection of a wide range of molecules including drug molecules simply based on the molecular spectral signature.^24–26^ Using SERS colloids, ultra-high detection sensitivity can be achieved, even down to the single-molecule level on the colloidal electromagnetic hotspots.^27,28^ However, the major challenge for SERS-based pharmacokinetics study (similar to other label-free SERS sensing techniques) is the poor reproducibility at low analyte concentrations^29^ and the spectral interference from the complex background matrix of biofluids.^30,31^ Therefore, to the best of our knowledge, very little work in SERS pharmacokinetic studies has achieved *in vitro* quantitative drug monitoring in live animals.^32,33^

Recently, our group has reported a new technique, digital colloid-enhanced Raman spectroscopy (dCERS), to circumvent the long-standing reproducibility issue in SERS.^34^ By employing single-molecule counting in the metallic colloidal suspension, we achieved robust quantification of various molecules at ultra-low concentrations down to 1 fM, ensuring controllable accuracy based on the Poisson rule. In particular, the error can be effectively reduced simply by accumulating the positive events. This method is cheap, rapid, easy to operate and widely applicable. Addressing spectral interference, molecules with nitriles, deuterium or alkynes, also termed bioorthogonal molecules, present innate advantages due to their characteristic SERS peaks in the spectral silent region (SR) (1800–2800 cm^−1^) where endogenous molecules show no Raman response.^35–37^ This feature ensures reliable identification and enhanced detectability of bioorthogonal molecules without interference from other background molecules from the samples in the fingerprint region (500–1800 cm^−1^).^38–40^ This intrinsic superiority has also been widely leveraged for cell imaging^41,42^ and identification of protein small-molecule-binding sites^43^ among the others.

Herein, we propose a pharmacokinetic detection technique for bioorthogonal drug molecules with nitriles, deuterium or alkyne groups in serum from live animals by using dCERS. This method has been first validated using 4-mercaptobenzonitrile (MBN) with a nitrile moiety using citrate-reduced silver (citrate–Ag) colloids for detection both in pure solution and in a complicated model mixture containing other similarly structured molecules without SR signatures. MBN was successfully quantified with the SR peak signal based on the counted positive events, adhering to the Poisson rule for quantification accuracy. For application, we demonstrated the method for the detection of erlotinib (ERL), a cancer treatment drug with a terminal alkyne to generate an SR signal. The sample pretreatment method has been optimized to be simple and effective with the aim to improve the detectability of erlotinib in serum. The calibration curve for erlotinib was established in advance in serum, and the serum pharmacokinetic profiles were then rapidly and successfully evaluated in animal studies and show consistent results with mass spectrometer and previous studies.^44–46^ In summary, the entire pharmacokinetic detection process is sensitive, cost-effective, rapid, and easy to operate, making it suitable for diverse fundamental research and clinical applications that demand high accuracy and minimizing cost and time. This method exhibits significant potential for broad use in the rapid quantification of bioorthogonal drug molecules.

## Results and discussion

**Fig. 1:**
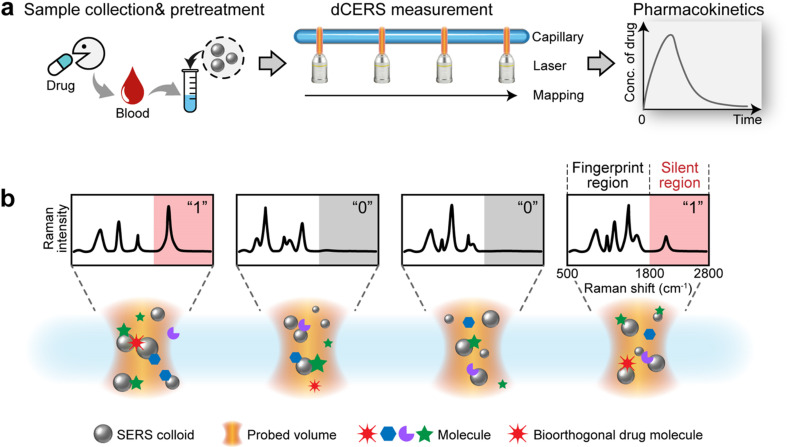
Schemes for the workflow of dCERS-based pharmacokinetic detection. (a) The blood samples are pretreated and mixed with the SERS colloids, followed by the dCERS measurement carried out in the pointwise scanning mode. The ratio of positive voxels to the total voxels is then computed to reflect the concentration of the drug molecules for the temporal monitoring. (b) The digitalization of each spectrum is performed by comparing the characteristic peak of the drug molecule in the silent region with a preset threshold. Specifically, the corresponding voxel is designated as positive (“1”) when the spectrum presents the target signal higher than the threshold, or as negative (“0”).

The workflow of dCERS pharmacokinetics consists of blood sampling, appropriate pretreatment, dCERS measurement and spectral analysis for drug molecule quantification using a pre-established calibration curve (Fig. 1a). Herein, the pretreatment method for blood samples should be tailored to enhance drug detectability on the SERS colloidal surface. Following the addition of SERS colloids into the pretreated samples, pointwise scanning is applied to acquire multiple spectra from the different voxels in the sample–colloid suspension. The signals can be counted with the Raman peak in the SR region when the target molecules reach a concentration exhibiting an average signal intensity of multiple spectra lower than the noise level. Each spectrum is then digitalized as positive (“1”) or negative (“0”) based on the intensity of a specific Raman peak compared to the preset threshold which judges the presence of the target drug molecules (see Fig. 1b). For the bioorthogonal drug molecules, their characteristic peak in the SR is leveraged, effectively avoiding spectral interference from other biological background molecules in the fingerprint region. The number of positive voxels is counted and the ratio of positive voxels (RPVs) to the voxels acquired in total is then used to reflect the concentration of the target molecules. Notably, for dCERS, the number of spectra should be predesigned according to the demanded quantification accuracy based on the Poisson rule.^34^ Before the measurement of real samples, a calibration curve should be established in advance using the standard samples with comparable background conditions for validity. Subsequently, the RPVs obtained from the real samples can be reliably converted to the concentration of the target molecules.

**Fig. 2:**
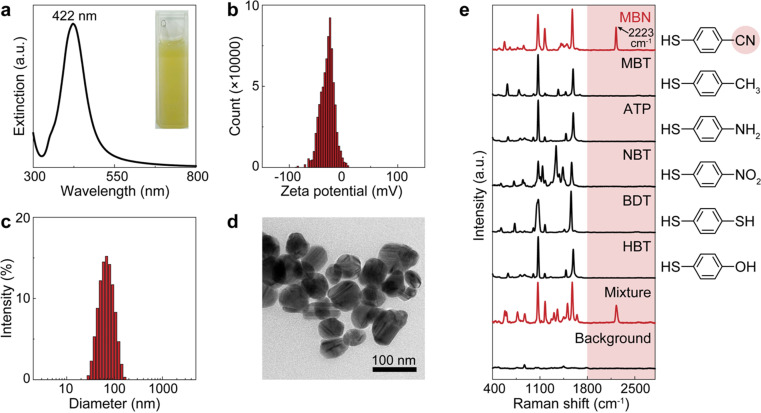
Characterization of the citrate–Ag colloids and the standard SERS spectra of the molecules used for model validation. (a) The extinction spectrum (inset: photo of the colloidal suspension), (b) the histogram of the zeta potential, (c) the histogram of the hydrodynamic diameter, and (d) the transmission electron microscopic image of the citrate–Ag colloids. (e) Normalized spectra of pure MBN, MBT, ATP, NBT, BDT, HBT, and the mixture of all the above molecules as well as the colloidal background. The molecular structures are provided on the right side. The red shades present the SERS spectral silent region and the C≡N moiety in MBN generating the silent-region signal.

For demonstration, we firstly used citrate–Ag colloids for SERS measurements, which are capable of label-free detection of a wide range of molecules including various drug molecules.^47,48^ This type of colloids exhibited single-molecule sensitivity as proved by the bi-analyte technique in previous studies and our demonstration,^34,49,50^ promising the single-molecule counting capability of dCERS. These colloids showed an extinction peak at 422 nm (Fig. 2a) and a zeta potential of −30.8 mV (Fig. 2b). Citrate–Ag colloids were monodispersed with a hydrodynamic diameter of about 72 nm (PDI = 0.196) (Fig. 2c), consistent with the transmission electron microscopic result (67 ± 14 nm) (Fig. 2d), ensuring a statistically uniform distribution throughout the detection system and a very low background signal (see the bottom of Fig. 2e). The stability, cost-effectiveness, and mass-producibility of citrate–Ag colloids make them suitable for long-term SERS measurements and broad applications. After the sample was mixed with the citrate–Ag colloids with a volume ratio of 1 : 9, the measurement was carried out on a confocal Raman system using a quartz capillary (I.D. = 1 mm) to hold 10 μL of the sample–colloid mixture. The pointwise scanning mode was used to acquire a set of spectra *via* a 10× objective lens and a step size of 10 μm was set to ensure the independency between adjacent voxels. To validate this concept, we used MBN as the target molecule, with a characteristic peak of 2223 cm^−1^ from the C≡N stretch in the SR,^51^ and other major peaks in the fingerprint region, *e.g.*, 1070 cm^−1^ from C–S stretching, 1170 cm^−1^ from aromatic in-plane C–H stretching and 1580 cm^−1^ from aromatic C=C stretching (see the top of Fig. 2e).^52,53^ The nitrile group offers a unique Raman feature in the SR for MBN, distinguishable from other structurally similar molecules including 4-methylbenzenethiol (MBT), 4-aminothiophenol (ATP), 4-nitrobenzenethiol (NBT), 1,4-benzenedithiol (BDT) and 4-hydroxybenzenethiol (HBT) (Fig. 2e). These five molecules have the same benzenethiol moiety as MBN but lack the nitrile group. Consequently, they all show overlapped vibrational peaks at the positions of 1070, 1170, and 1580 cm^−1^ in the fingerprint region due to the common benzenethiol moiety, but no peak in the SR.

**Fig. 3:**
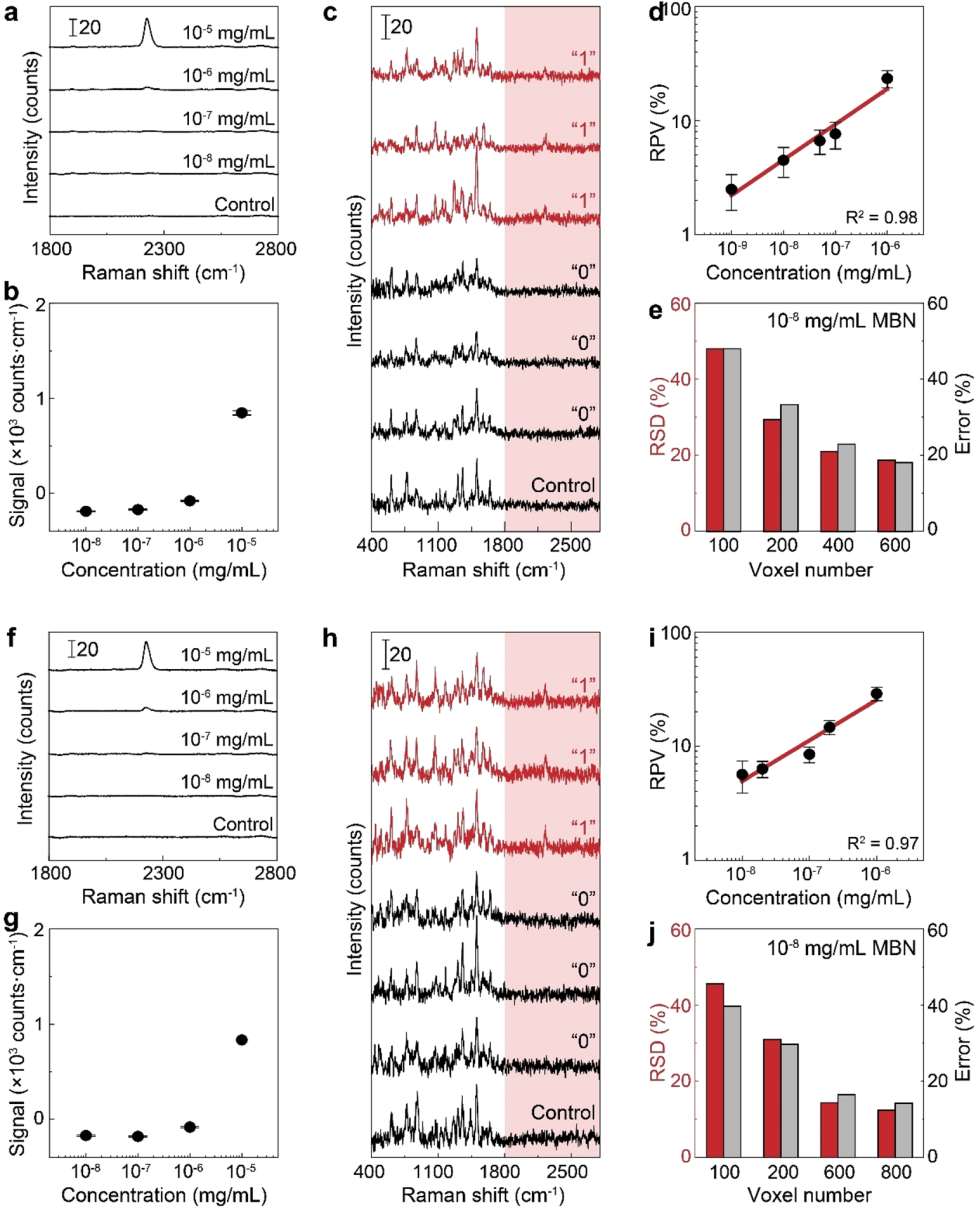
Quantification of MBN. (a)–(e) The pure MBN solution. (a) The mean spectra of MBN–ethanol solution (10^−5^ to 10^−8^ mg mL^−1^) and the control (ethanol without MBN). (b) The peak signal from the mean spectra over all voxels. Each data point is shown by mean and the standard deviation is calculated from 3 measurements. (c) Typical positive (“1”) and negative (“0”) spectra of MBN and a typical spectrum of the control sample. (d) Calibration curve of MBN by dCERS. (e) The voxel number-dependent quantification accuracy at 10^−8^ mg per mL MBN. (f)–(j) The mixture of MBN and other 5 molecules. (f) The mean spectra of the mixture with different concentrations of MBN and the control (the other 5 molecules except MBN in ethanol). (g) The peak signal from the mean spectra with the error bar indicating the standard deviation (*n* = 3). (h) Typical positive (“1”) and negative (“0”) spectra of MBN in the mixture and a typical control spectrum. (i) dCERS calibration curve of MBN and (j) the voxel number-dependent quantification accuracy at 10^−8^ mg per mL MBN in the mixture. In panels (e) and (j), the relative standard deviation (RSD, red bar) is calculated as the ratio of the standard deviation to the mean of the positive counts from three measurements, consistent with the Poisson estimation (
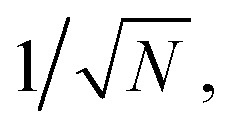
 gray bars) from the mean positive counts (*N*).

To verify the use of the SR peak for dCERS quantification, we prepared a series of pure MBN solution (solvent: ethanol) at the concentrations ranging from 10^−5^ to 10^−8^ mg mL^−1^ and mixed with the citrate–Ag colloids, using the 638 nm incident laser (power = 12.67 mW) and 0.1 s of acquisition time for measurement. For each sample, 200 spectra were acquired. The MBN signal of each spectrum was calculated by using the integral area from the spectral window of 2205–2255 cm^−1^ covering the targeted SR peak of 2223 cm^−1^ (*A*_SR_) subtracted by 3 times the integral area from the noise window of 2400–2450 cm^−1^ (*A*_noise_) where there were no SERS peaks, *i.e.*, signal_MBN_ = *A*_SR_ − 3 × *A*_noise_. Herein, the integral area was adopted to reduce the estimation error caused by the increasing noise level as the wavenumber in the silent region due to the instrumental factor. As seen from the mean signal of 200 spectra of each sample (Fig. 3a and b), the SR peak of MBN is clearly distinguishable at 10^−5^ mg mL^−1^, while at concentrations of 10^−6^ mg mL^−1^ and below, it becomes either below the noise level or non-discriminable from the control sample (ethanol without MBN). Therefore, in these low concentrations (10^−6^ to 10^−8^ mg mL^−1^), each spectrum was used and digitalized as positive (“1”) when signal_MBN_ > 0, or as negative (“0”). It is worth mentioning that, though some peaks in the fingerprint region might overlap with background molecular peaks such as 1070 cm^−1^ of MBN probably immersed in the dual peaks of 1040 cm^−1^ (C–C–O stretching) and 1080 cm^−1^ (CH_3_ rocking) present in ethanol,^54^ the SR peak unique to MBN can be confidently distinguished from background spectral signatures (Fig. 3c). As a result, the relationship between the RPV, computed as the percentage of positive voxels among the total measured voxels, and the concentration of MBN can be well fitted by a linear line on the log–log scale (Fig. 3d). In this sense, dCERS is obviously superior to the analog method at low concentrations given that the frequency of signals to appear above a specific threshold should be a certain probability at any molecular concentration owing to the statistically uniform distribution of hotspots across the probed volumes. Therewith, digitalization of the intensity magnitude based on a predetermined standard can effectively eliminate the signal intensity fluctuation caused by the heterogeneous colloidal hotspots and molecular behavior. It can also be found that the measurement error is continuously increasing when the target molecule concentration is decreased (namely, the decrease of the positive events). This exactly means that the counting-based quantification follows the Poisson distribution, leading to the quantification accuracy well controlled by the Poisson square root law (
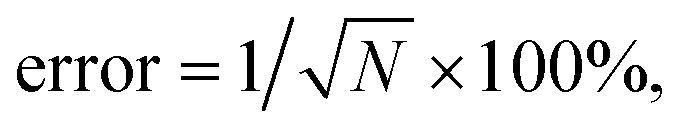
 herein, *N* is the number of positive voxels).^34^ Typically, the error can be reduced by accumulating more voxels at ultralow molecular concentrations. For example, even at 10^−8^ mg mL^−1^, though the relative standard deviation among 3 measurements was relatively large (48%) when only 100 voxels were acquired for each measurement, it can be effectively reduced to 18.7%, when the number of totally acquired voxels per measurement was increased to 600 so that the number of positive voxels was accumulated from 4 to 30 (Fig. 3e).

To mimic a real biological system with numerous structurally similar molecules, we chose to mix MBN with the above-mentioned five molecules, *i.e.*, MBT, ATP, NBT, BDT and HBT as the background molecules. Obviously, a molecular mixture with the above six molecules may generate overlapping peaks in the fingerprint region (*i.e.*, 1070, 1170 and 1580 cm^−1^) while the peak in the SR should only originate from MBN (Fig. 2e). Thereafter, we generated a series of mixtures with different concentrations of MBN (final concentration: 10^−5^ to 10^−8^ mg mL^−1^) and the other 5 background molecules (10^−8^ mg mL^−1^). As a result, when the concentration of MBN is lower than 10^−6^ mg mL^−1^, the analog intensity at the SR peak of MBN is not strong enough for reliable quantification (Fig. 3f and g), while by examining every single spectrum, the SR peak was still discriminate from the noise at some voxels (Fig. 3h). In such a complicated mixture, the advantage of using the SR peak is more apparent that only according to their SR peaks can the bioorthogonal molecules be identified reliably with no spectral interference from other molecules, even if the peaks overlapping in the fingerprint region became more serious given the coexisting structurally similar molecules. By counting the positive voxels at each concentration, we succeeded in building a linear calibration curve on the log–log scale in the six-molecule mixture (Fig. 3i). This curve exhibited a similar slope and bias to that obtained in the pure solution, indicating that there is a negligible influence on the detectability of target molecules. Actually, when the overall composition of the background matrix remains roughly unchanged, this influence might be a constant factor, *i.e.*, reduced number of available hotspots,^55,56^ which will be discussed in detail in the following part. Besides, the quantification accuracy in the mixture still followed the Poisson square root with the accumulated positive voxels determining the error. As shown in Fig. 3j, the relative standard deviation at 10^−8^ mg per mL MBN was reduced from 46% to 12% when measured voxels were increased from 100 to 800 voxels.

**Fig. 4:**
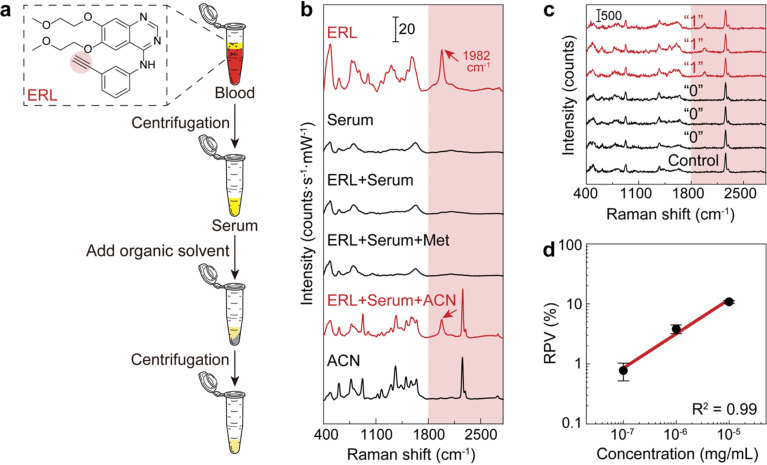
The calibration curve of ERL in serum. (a) Pretreatment workflow of the blood sample. (b) SERS spectra of pure ERL, serum, ERL-doped serum (ERL + serum), the supernatant of ERL-doped serum after adding methanol (ERL + serum + Met) and acetonitrile (ERL + serum + ACN) for deproteinization, and pure acetonitrile (ACN). (c) Typical positive (“1”) and negative (“0”) spectra from the ERL-doped acetonitrile-pretreated serum sample and the spectrum from an acetonitrile-pretreated serum sample without ERL (control). (d) The calibration curve of ERL in serum with acetonitrile pretreatment (error bar: standard deviation, *n* = 3).

The above results inspire us to further detect the concentration of bioorthogonal drugs in the serum with the dCERS technique. For the demonstration of dCERS-based pharmacokinetic detection, we used ERL as the targeted drug molecule, which is the first-generation epidermal growth factor receptor (EGFR) tyrosine kinase inhibitor for the treatment of EGFR exon 19 deletions (ex19del) or exon 21 L858R substitution mutated non-small cell lung cancer (NSCLC).^57,58^ ERL has a terminal alkynyl moiety (Fig. 4a), exhibiting a bioorthogonal characteristic SERS peak at 1982 cm^−1^ for C≡C stretching with strong C≡C–Ag surface interaction when incubated with citrate–Ag colloids.^59^ Other major peaks in the fingerprint region include 775 cm^−1^ for quinazoline group aromatic ring out-of-plane deformation, 993 cm^−1^ for phenyl ring deformation, 1305 cm^−1^ for quinazoline group C=N stretching and CC stretching, and 1582 cm^−1^ for phenyl ring CC stretching^59^ (see the top of Fig. 4b).

In order to perform the dCERS detection of ERL in the real biological samples with a complex background matrix such as blood,^60^ given that abundant molecules coexist in the solution with drastically variable properties, appropriate pretreatment methods should be selected to improve the SERS detectability. In the serum, a major obstacle that prevents small molecules from being detected is the presence of abundant proteins, which may generate the protein corona effect on the surface of the metallic colloids.^61–63^ Herein, we compared the detectability of ERL in rat serum by using two common protein precipitation organic solutions for deproteinization, *i.e.*, methanol and acetonitrile, in the pretreatment process (Fig. 4a). As shown in Fig. 4b, for ERL in serum without pretreatment, the spectrum presented hardly any observable peaks from ERL, which was almost the same as the spectrum of the serum without ERL. This indicated that the small drug molecules were possibly prevented from getting close to the electromagnetic hotspots of the colloids by the proteins. After the addition of the methanol to the serum with ERL in a ratio of 3 : 1 (vol/vol) according to the common practice^64^ for protein denaturation,^65,66^ there were still no obvious changes. In comparison, the SR peak of ERL clearly emerged when using acetonitrile in the same ratio as methanol probably because acetonitrile is more effective at protein removal than methanol.^67^ Though acetonitrile also shows several peaks in the fingerprint region and one in the silent region (2260 cm^−1^) due to the C≡N stretching,^68,69^ the SR peak of ERL at 1982 cm^−1^ does not overlap with any of them, ensuring reliable identification of ERL (see Fig. 4b). Therefore, we decided to use acetonitrile for deproteinization as the pretreatment to increase the detectability of ERL without spectral interference. Actually, other organic solvents can also be flexibly used for the purpose of deproteinization (Fig. S1†) as long as there is no spectral interference with the drug molecule and adequate deproteinization efficiency for sensitive detection.

Subsequently, we prepared serum samples with varied concentrations of ERL and applied the same pretreatment method (acetonitrile : serum = 3 : 1 vol/vol). Measurements were conducted with a 532 nm incident laser (power = 19.9 mW) and the acquisition time of 1 s for higher detectability (Fig. S2†). The integral area from 1930 to 2030 cm^−1^ after noise subtraction was applied to reflect the signal of ERL without interference from the background matrix and acetonitrile. For concentrations lower than 10^−5^ mg mL^−1^, the signal fell below the noise level (Fig. S3†), rendering it unsuitable for analog quantification. Therefore, we digitalized each spectrum for dCERS (Fig. 4c). By counting the number of positive voxels, we built a calibration curve in the serum, exhibiting a linear relationship on the log–log scale between RPV and ERL concentration (Fig. 4d). It is worth mentioning that compared with the dCERS measurement conducted in pure solution, the detectability of erlotinib in serum was much lower because there are a large number of biomolecules (*e.g.*, metabolites) existing in the serum even after pretreatment, which may occupy a certain proportion of the colloidal surface, therewith reducing the available hotspots for erlotinib (Fig. S4†). Nevertheless, given the fact that the overall composition of serum was similar, the available hotspots were reduced by a constant factor regardless of the concentration of the target molecules, leading to the calibration curve only shifted from that in pure solution by a certain factor. This guaranteed the validity of the pre-calibration, and in the practical sense, it is more straightforward to calibrate in the intended detection system.^70,71^ Apart from the pretreatment mentioned above, other methods including extraction can also be leveraged to further diminish the influence of coexisting biomolecules thus to increase the detection efficiency of the target drug molecules. Besides, the total number of voxels accumulated was gradually increased with the lowering concentration for the validity of the calibration curve (see the Methods section for details). For the following pharmacokinetic application, since the calibration curve is established with the final concentration of ERL in the sample–colloid suspension, the actual concentration of ERL in blood should be multiplied by 40 times to recover from the dilution by acetonitrile and the Ag colloids. Nevertheless, 10^−7^ mg mL^−1^ (equal to 4 ng per mL ERL in blood) is slightly lower than the current limit of detection by conventional LC-MS^72^ and even lower concentrations and higher accuracy are possible to be achieved by accumulating more voxels according to the Poisson statistics.

**Fig. 5:**
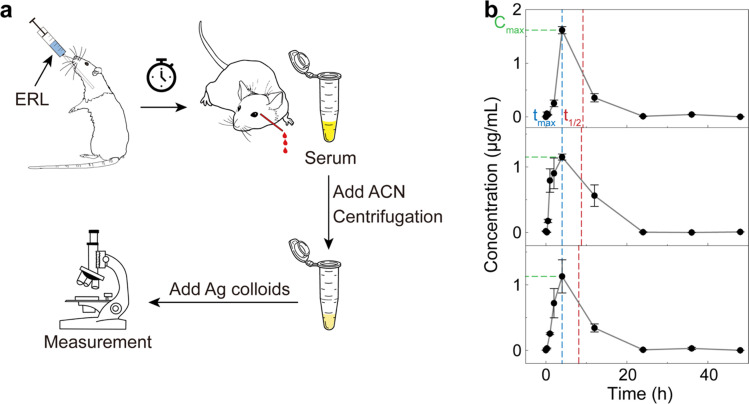
Pharmacokinetic detection of ERL using rats. (a) Schematic workflow. ERL was orally administered to rats, followed by blood collection at a series of time points. Serum was then obtained and pretreated with acetonitrile. The supernatant was collected after centrifugation and mixed with the citrate–Ag colloids for the subsequent SERS measurement. (b) The time-dependent concentration of ERL in rat blood computed based on the preestablished calibration curve (Fig. 4d). Error bar: standard deviation from 3 measurements at each time point. Pharmacokinetic parameters of *C*_max_, *t*_max_ and *t*_1/2_ are indicated by the green, blue and red dashed lines, respectively.

To simulate the pharmacokinetic detection of ERL in blood (Fig. 5a), we orally administered a dose of 20 mg per kg body weight ERL to male Wistar rats (∼250 g, *n* = 3). The whole blood was withdrawn from the retro-orbital plexus of the rats at a series of time points from 0 (right after drug administration) to 48 hours post-administration. The serum was obtained from the blood using standard protocols,^73–76^ followed by the acetonitrile pretreatment for deproteinization as described above. After the addition of citrate–Ag colloids, three measurements (400 voxels per measurement) were implemented for each sample for accuracy. The RPV of ERL based on the SR peak signal was then calculated for each measurement, and then converted to the concentration based on the pre-established calibration curve in Fig. 4d.

The obtained ERL concentration–time profile (Fig. 5b) revealed similar pharmacokinetic behavior in the three rats. Specifically, at 0 hour (the starting point), the ERL concentration was approximately 0 since the drug had not yet been absorbed. Subsequently, a sharp increase in blood ERL concentration occurred due to the rapid absorption, reaching peak concentration around 4 hours post-administration, which may be slightly different from the experimental value in some pervious studies^44^ due to the relatively few sampling points. Further metabolism led to ERL elimination from the blood, with concentrations returning to around 0 after 24 hours, signifying mostly complete drug excretion. Pharmacokinetic parameters, including maximum serum concentration (*C*_max_), time to reach the maximum concentration (*t*_max_) and half-life (*t*_1/2_), were calculated as 1.3 ± 0.28 μg mL^−1^, 4 ± 0 hours, and 4.7 ± 0.5 hours, respectively, based on the noncompartmental model. These results, though slightly different due to variations in pretreatment methods and individual rat differences, have been verified by liquid chromatography-tandem mass spectrometry (LC-MS/MS) under conventional protocols (see Fig. S5† and Methods for details) and found comparable to previous studies,^44–46^ further validating our methods for pharmacokinetic detection.

As demonstrated in the above application, dCERS-based pharmacokinetic detection of the bioorthogonal drug molecules is fast, requires only simple pretreatment and low-cost instruments throughout the whole procedure. Since portable Raman devices are easily available at present, they further shed light on point-of-care testing for real-time pharmacodynamic monitoring and personalized pharmacotherapy. Using the dCERS technique, we demonstrated an accuracy-controllable quantitative *ex vivo* pharmacokinetic detection, with sensitivity down to the nanomolar range (possibly even lower) in comparison to the micromolar level as demonstrated in other literature studies.^77^ More importantly, dCERS is capable of sensitive and accurate quantification even in a complicated biological background as long as a calibration curve is established in advance in the comparable system with the measurement parameters including the total number of measurement voxels predesigned according to the practical demands.

## Conclusion

In conclusion, we revealed that bioorthogonal drug molecules can be reliably quantified based on the SR signature by dCERS. Using MBN as the target molecule with a unique SR peak, our method exhibited superior performance to the analogous method in terms of LOD, reproducibility at low concentrations, and minimal interference from background molecules. The counting-based SERS quantification displayed accuracy in line with the Poisson rule, further improvable with the accumulation of positive voxels. In real applications, we successfully applied this method to the quantitative pharmacokinetic detection of erlotinib, a bioorthogonal drug molecule, in animal studies, demonstrating higher sensitivity, simple and rapid pretreatment and measurement (less than 30 minutes in total), as well as lower cost (only several dollars) than conventional LC-MS. Particularly, as it may be sometimes difficult to obtain a calibration curve owing to the complex background composition and properties, appropriate pretreatment should be applied to the specific detection system in order to reduce the background interference and increase the detectability of the target drug molecules, such as using acetonitrile for serum deproteinization as in this work, for reliable quantification. Given the current prominence of bioorthogonal drug molecules in research, our method paves a promising route for pharmacokinetics and other essential clinical tests, offering fast, accurate, and sensitive drug quantification in biofluids.

## Methods

### Materials and instrumentation

Trisodium citrate (TSC, AR, 99.8%), silver nitrate (AgNO_3_, AR, 99.8%), 4-mercaptobenzonitrile (MBN, 95%), 1,4-benzenedithiol (BDT, GC, 98%), 4-hydroxybenzenethiol (HBT, 97%), 4-aminothiophenol (ATP, GC, 98%), 4-methylbenzenethiol (MBT, 98%), 4-nitrobenzenethiol (NBT, 95%), methanol (99.9%) and acetonitrile (GC, 99.9%) were obtained from Aladdin (Shanghai, China). Ethanol (AR, 99.7%) was purchased from Sinopharm (Shanghai, China). Erlotinib (ERL, HPLC, 98%) and carboxymethyl cellulose (CMC, 0.5%) were bought from Sigma-Aldrich (Shanghai, China) and Yuanye Bio-Technology (Shanghai, China), respectively. All materials were used as received without any further purification. Ultrapure water (18.0 MΩ) was used for all experiments.

The extinction spectra of the SERS colloids were measured using a UV1900 UV-vis spectrophotometer (Aucybest, Shanghai, China). The hydrodynamic diameter and the zeta potential of the colloids were obtained using a Zetasizer Nano ZSP (Malvern, UK). A JEM-2100F transmission electron microscope (JEOL, Tokyo, Japan) was used to characterize the morphology of the colloids.

### Synthesis of the SERS colloids

The citrate-reduced Ag colloids were synthesized according to Lee and Meisel's method^78^ with slight modification. In brief, 12.3 mg AgNO_3_ was dissolved in 100 mL ddH_2_O and brought to a boil, followed by the addition of 2 mL of 1% TSC. The mixture was kept boiling for another 1 hour and then cooled to room temperature under constant stirring. The product was stored at 4 °C without exposure to light for later use.

### Rat serum collection

Animal experiments were performed in compliance with the Bioethics Committee of School of Biomedical Engineering, Shanghai Jiao Tong University (No. 2023025). All animal housing and experiments were conducted in accordance with the ethical standards of the institute. The rats (male Wistar rats, 7 weeks, about 250 g) were purchased from Shanghai SLAC Laboratory Animal Co. LTD (Shanghai, China). For the pharmacokinetic study, the rats were orally gavaged with 20 mg per mL ERL in 0.5% CMC at the dose of 1 mL per kg body weight after 12 hour fasting. Blood (0.3–0.5 mL) was withdrawn at desired timepoints from the rats at the retro-orbital plexus using a plain capillary tube and allowed to stand still for 30 min. Then, the blood was centrifugated at 2500 rpm for 10 min to obtain the serum.

### Sample preparation

To construct the 6-molecule mixture, MBT, ATP, NBT, BDT and HBT (final concentration: 0.01 ng mL^−1^) were mixed with different concentrations of MBN in ethanol. A corresponding control sample was prepared at the same composition but without MBN. To obtain the calibration curve of ERL in blood, ERL was first dissolved in methanol at the concentration of 1 mg mL^−1^, then added in the serum to reach a series of different concentrations, followed by ultrasonication for complete dissolution. For protein precipitation, the serum including the ERL-doped ones was further centrifuged at 12 500 rpm for 10 min after methanol or acetonitrile was added in the serum at a ratio of 3 : 1. The supernatant was collected for measurement.

### SERS measurement

Before measurement, all the samples were mixed with the citrate–Ag colloids at the ratio of 1 : 9 and then incubated for 10 min. 10 μL sample–colloid mixture was then injected into a quartz capillary (I.D.: 1 mm, O.D.: 2 mm) and measured using a confocal Raman system (Horiba, XploRA INV) *via* a 10× objective lens in the pointwise scanning mode (step size: 10 μm). The incident laser wavelengths used are 532 nm (power: 19.9 mW) and 638 nm (power: 12.67 mW) as indicated above.

### Data analysis

All the spectra were first preprocessed to remove the cosmic rays and the backgrounds on the LabSpec 6 software (Horiba Scientific). The signal of the target molecule was calculated for each spectrum on MATLAB® R2022b from the integral area of the spectral window containing the characteristic SERS peak of the target molecule subtracted by 3 times of the integral area of the noise window. For digitalization, the spectrum was designated as positive (“1”) if the target signal was larger than 0, otherwise as negative (“0”). For MBN, the target window was selected as 2205–2255 cm^−1^ and the noise window was 2400–2450 cm^−1^. For ERL, the target window was 1930–2030 cm^−1^ and the noise window was 2700–2800 cm^−1^. The ratio of positive voxel was computed as the percentage of positive spectra among all the acquired spectra in one measurement. For the validity of the calibration curve of ERL in serum, the measured voxel number per measurement was 200 at 10^−5^ mg per mL ERL, 400 at 10^−6^ mg mL^−1^ and 600 at 10^−7^ mg mL^−1^. The noncompartmental analysis was performed to obtain the pharmacokinetic parameters with the SimBiology Model Analyzer app on MATLAB® R2022b.

### LC-MS/MS measurement of erlotinib in rat serum

Erlotinib quantification was performed using an Agilent 1290 Infinity UHPLC system coupled with an Agilent 6495 Triple Quadrupole MS equipped with an electrospray ionization (ESI) source (Agilent, Palo Alto, USA). Data acquisition was performed *via* MassHunter software (Agilent, Palo Alto, USA).

Chromatographic separation was performed using an ACQUITY UPLC BEH C18 column (50 mm × 2.1 mm, 1.7 μm) from Waters, USA. The mobile phase consisted of water (containing 0.1% formic acid) for A and acetonitrile (containing 0.1% formic acid) for B. The flow rate was 0.5 mL min^−1^. The gradient program was as follows: 0–0.4 min, 30% B; 0.4–1.3 min, 30–95% B; 1.3–1.7 min, 95% B; 1.7–1.8 min, 95–30% B; and 1.8–2.2 min, 30% B. The column temperature was maintained at 40 °C, the sample chamber temperature was set at 10 °C. The sample injection volume was 2 μL.

The MS was operated in the positive ion mode. The optimized tuning parameters were as follows: the gas temperature was set to 200 °C, gas flow was 14 L min^−1^, nebulizer pressure was 20 psi, sheath gas temperature was 250 °C, sheath gas flow was 11 L min^−1^, the fragmentor was set at 380 eV, and capillary voltage was 4000 V. The optimized multiple reaction monitoring (MRM) transitions were *m*/*z* 394.3 → *m*/*z* 277.8 for erlotinib and *m*/*z* 455.3 → *m*/*z* 165.2 for the internal standard (IS, verapamil). The collision energy (CE) applied for the two was 40 eV. The dwell time for each transition was set to 200 ms.

To prepare calibration samples of erlotinib in the serum, the concentrations of erlotinib in the calibration standards were 1, 2, 4, 10, 40, 100, 400, 1000, and 2000 ng mL^−1^. An aliquot of 10 μL calibration standards and serum sample was protein-precipitated with 90 μL methanol and acetonitrile (1 : 1, vol/vol) containing 20 ng per mL verapamil. The mixture was vortexed for 5 min and centrifuged at 4000 rpm for 15 min. 50 μL supernatant was mixed with 70 μL water. The mixture was injected for LC-MS/MS analysis.

## Author contributions

J. Y. conceived the research. Z. H. synthesized silver particles and performed the SERS measurement. X. B. analyzed the data and wrote the draft. Z. L. reviewed other relevant papers. W. H. performed the MS measurement. All authors wrote and revised the manuscript. X. B. and Z. H. contributed equally to this work.

## Conflicts of interest

The authors declare no conflicts of interest.

## Supplementary Material

SC-015-D4SC02553A-s001

## Data Availability

The authors declare that the data supporting the findings of this study are available within the paper and its ESI files.† All relevant data are available from the corresponding author on request.
