# Supplementary material for: Digital colloid-enhanced Raman spectroscopy for the pharmacokinetic detection of bioorthogonal drugs
Source: Chem Sci. 2024 Aug 2;15(34):13998–4008. doi: 10.1039/d4sc02553a (PMC11320124; doi:10.1039/d4sc02553a)
Supplement: SC-015-D4SC02553A-s001 [file SC-015-D4SC02553A-s001.pdf]

Supplementary Information (SI) for Chemical Science.
This journal is © The Royal Society of Chemistry 2024

Supporting Information

Digital colloid-enhanced Raman spectroscopy for the pharmacokinetic detection of bioorthogonal drugs

Xinyuan Bi,[Δ,†] *Zhicheng He,*[Δ,†] *Zhewen Luo,*[†] *Wensi Huang,* [†,§] *Xingxing Diao,*[§, £] *and Jian Ye*[*,†,δ,θ,β]

[†]State Key Laboratory of Systems Medicine for Cancer, School of Biomedical Engineering, Shanghai Jiao Tong University, Shanghai, 200030, P. R. China

[§]Shanghai Institute of Materia Medica, Chinese Academy of Sciences, Shanghai, 201210, P. R. China

[£]University of Chinese Academy of Sciences, Beijing, 100049, P. R. China

[δ]Institute of Medical Robotics, Shanghai Jiao Tong University, Shanghai, 200240, P.R. China

[θ]Shanghai Key Laboratory of Gynecologic Oncology, Ren Ji Hospital, School of Medicine, Shanghai Jiao Tong University, Shanghai, 200127, P.R. China

[β]Sixth People's Hospital, School of Medicine, Shanghai Jiao Tong University, Shanghai, 200233, P.R. China

Footnotes

[Δ] These authors contributed equally.

[*]To whom correspondence should be addressed. E-mail: yejian78@sjtu.edu.cn

Contents

Fig. S1. Serum pretreatment by organic solvents for deproteinization. SERS spectra of erlotinib-doped serum (ERL+Serum), the supernatant of erlotinib-doped serum after isopropanol for deproteinization (ERL+Serum+Isopropanol), the supernatant of erlotinib-doped serum after ethanol for deproteinization (ERL+Serum+Ethanol), and the supernatant of erlotinib-doped serum after acetone for deproteinization (ERL+Serum+Acetone). The spectral silent region (SR) (1800 – 2800 cm^{-1}) is indicated by the red shade and the characteristic peak is indicated by a red arrow at 1982 cm^{-1}. 3

Fig. S2. SERS spectra of Erlotinib solution (concentration: 10^{-5} mg/mL) by using 532 nm (red) and 638 nm (black) of incident laser wavelengths. 4

Fig. S3. Quantification of Erlotinib in the serum by analog method. (a) The mean spectra of the serum samples with different concentrations of Erlotinib (10^{-4} – 10^{-7} mg/mL) and without Erlotinib (control). (b) The peak signal from the mean spectra over all voxels. Each data point is shown by mean and the standard deviation is calculated from 3 measurements. ($Peak\ signal = AreaErlotinib\ SR\ peak - 3 \times Areanoise$)............5

Fig. S4. Comparison between the calibration curve of ERL in pure water solution (circle) and in serum using acetonitrile for deproteinization (square, also in **Fig. 4d**). The slopes (k) of the calibration curves obtained by linear fitting on the log-log scale ($logRPV = klogConcentration + b$) are 0.55 and 0.57, respectively. 6

Fig. S5. Verification of SERS pharmacokinetic results by LC-MS/MS. Each datapoint is demonstrated by the mean erlotinib concentration in the serum samples collected from 3 rats and the error bar indicates the standard deviation (n = 3). 7

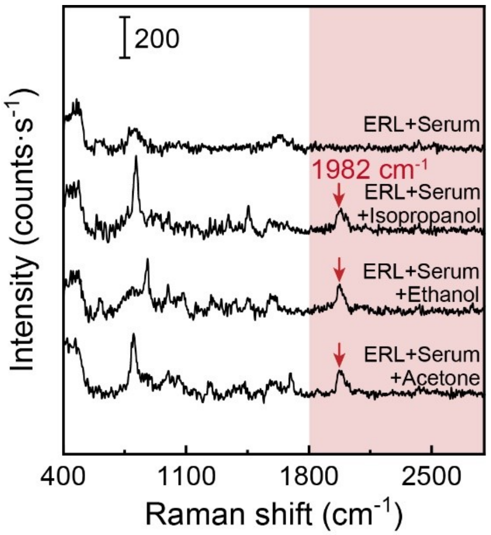

Fig. S1. Serum pretreatment by organic solvents for deproteinization. SERS spectra of erlotinib-doped serum (ERL+Serum), the supernatant of erlotinib-doped serum after isopropanol for deproteinization (ERL+Serum+Isopropanol), the supernatant of erlotinib-doped serum after ethanol for deproteinization (ERL+Serum+Ethanol), and the supernatant of erlotinib-doped serum after acetone for deproteinization (ERL+Serum+Acetone). The spectral silent region (SR) (1800 – 2800 cm^{-1}) is indicated by the red shade and the characteristic peak is indicated by a red arrow at 1982 cm^{-1}.

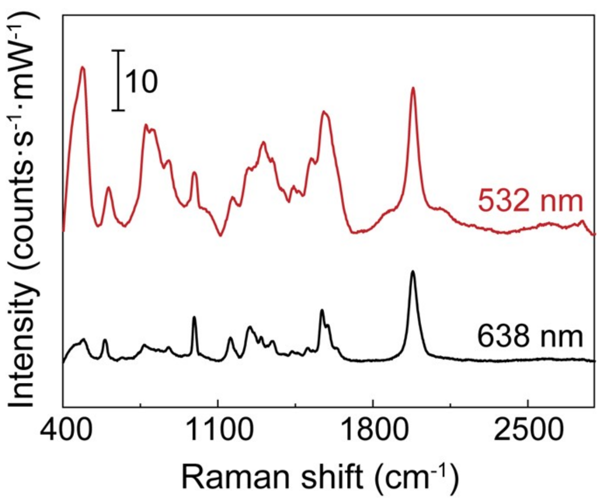

Fig. S2. SERS spectra of Erlotinib solution (concentration: 10^{-5} mg/mL) by using 532 nm (red) and 638 nm (black) of incident laser wavelengths.

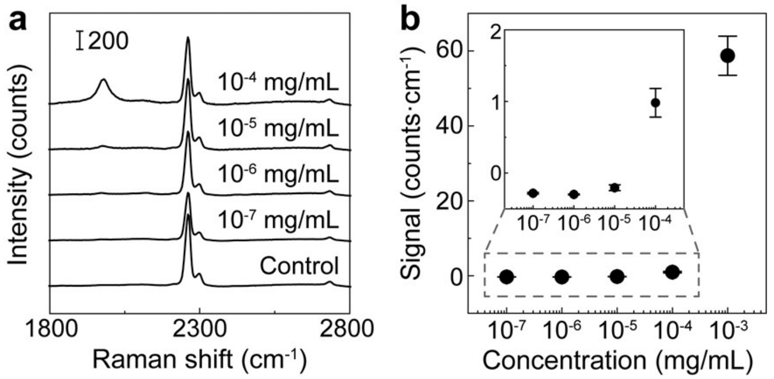

Fig. S3. Quantification of Erlotinib in the serum by analog method. (a) The mean spectra of the serum samples with different concentrations of Erlotinib (10^{-4} – 10^{-7} mg/mL) and without Erlotinib (control). (b) The peak signal from the mean spectra over all voxels. Each data point is shown by mean and the standard deviation is calculated from 3 measurements. ($Peak\ signal = Area_{Erlotinib\ SR\ peak} - 3 \times Area_{noise}$)

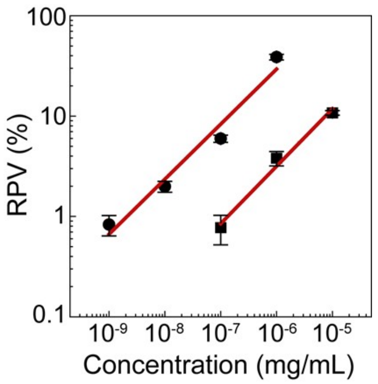

Fig. S4. Comparison between the calibration curve of ERL in pure water solution (circle) and in serum using acetonitrile for deproteinization (square, also in **Fig. 4d**). The slopes (k) of the calibration curves obtained by linear fitting on the log-log scale ($\log RPV = k\log Concentration + b$) are 0.55 and 0.57, respectively.

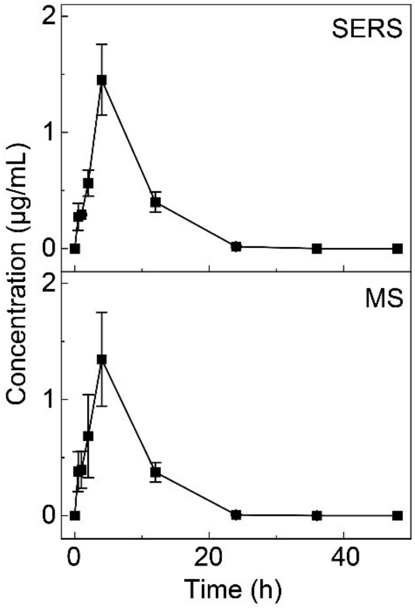

Fig. S5. Verification of SERS pharmacokinetic results by LC-MS/MS. Each datapoint is demonstrated by the mean erlotinib concentration in the serum samples collected from 3 rats and the error bar indicates the standard deviation (n = 3).
